# Uncorrelated Neural Firing in Mouse Visual Cortex during Spontaneous Retinal Waves

**DOI:** 10.3389/fncel.2017.00289

**Published:** 2017-09-20

**Authors:** Matthew T. Colonnese, Jing Shen, Yasunobu Murata

**Affiliations:** Department of Pharmacology and Physiology, Institute for Neuroscience, The George Washington University Washington, DC, United States

**Keywords:** visual cortex, development, spontaneous activity, synchronization, retinal wave, oscillation, spindle-burst

## Abstract

Synchronous firing among the elements of forming circuits is critical for stabilization of synapses. Understanding the nature of these local network interactions during development can inform models of circuit formation. Within cortex, spontaneous activity changes throughout development. Unlike the adult, early spontaneous activity occurs in discontinuous population bursts separated by long silent periods, suggesting a high degree of local synchrony. However, whether the micro-patterning of activity within early bursts is unique to this early age and specifically tuned for early development is poorly understood, particularly within the column. To study this we used single-shank multi-electrode array recordings of spontaneous activity in the visual cortex of non-anesthetized neonatal mice to quantify single-unit firing rates, and applied multiple measures of network interaction and synchrony throughout the period of map formation and immediately after eye-opening. We find that despite co-modulation of firing rates on a slow time scale (hundreds of ms), the number of coactive neurons, as well as pair-wise neural spike-rate correlations, are both lower before eye-opening. In fact, on post-natal days (P)6–9 correlated activity was lower than expected by chance, suggesting active decorrelation of activity during early bursts. Neurons in lateral geniculate nucleus developed in an opposite manner, becoming less correlated after eye-opening. Population coupling, a measure of integration in the local network, revealed a population of neurons with particularly strong local coupling present at P6–11, but also an adult-like diversity of coupling at all ages, suggesting that a neuron’s identity as locally or distally coupled is determined early. The occurrence probabilities of unique neuronal “words” were largely similar at all ages suggesting that retinal waves drive adult-like patterns of co-activation. These findings suggest that the bursts of spontaneous activity during early visual development do not drive hyper-synchronous activity within columns. Rather, retinal waves provide windows of potential activation during which neurons are active but poorly correlated, adult-like patterns of correlation are achieved soon after eye-opening.

## Introduction

Connectivity during development is achieved by synapse formation under the control of molecular guidance cues, and modification of these synapses by neural activity (Katz and Shatz, 1996). Activity influences circuit formation by coordinating firing between pre and post-synaptic neurons (Zhang and Poo, 2001; Kirkby et al., 2013). Thus, the degree of synchronization is a critical characteristic that determines the mechanisms of activity-dependent development (Butts and Kanold, 2010), but how synchronization and local network interactions change between critical epochs of development, for example between early spontaneously generated activity and later sensory experience, is poorly understood, particularly within single columns in isocortex.

Two broad models of circuit formation exist. In the “refinement” model, early hyper-connectivity caused by random synaptogenesis is replaced during a period of refinement with mature connections (Purves and Lichtman, 1980). In the “constructionist” model, correct connections emerge gradually without a period of hyper-connectivity (Quartz and Sejnowski, 1997). These models are not mutually exclusive and can reference anatomical connectivity as well as functional connectivity. In the visual system there is clear anatomical and functional refinement of visuotopic connectivity (Huberman et al., 2008). The rules guiding formation of columnar connectivity in cortex, within a single topographic location, are more poorly understood. One possibility (refinement model) is that all neurons respond synchronously to low-frequency maps such as topography before network fractionation into local microcircuits, such as for orientation or direction selectivity (White and Fitzpatrick, 2007; Butts and Kanold, 2010). Such synchronization would be driven by anatomical hyper-connectivity, but also by circuit properties such as weak inhibition, excitatory GABA_A_ currents, long channel decay-time, abundant electrical connectivity, and high neuron excitability which increase synchronization and reduce the specificity of neuronal responses (Blankenship and Feller, 2010; Cossart, 2011; Dehorter et al., 2012). A constructionist perspective would predict that synchronization emerges gradually and in parallel with the specialization of neuronal function by cell class and/or response properties (Erwin and Miller, 1998; Crowley and Katz, 2002).

A substantial body of evidence suggests early cortical development is governed by a refinement model. The period of circuit formation is a time of dramatic shift in the patterns of activity (Khazipov et al., 2013; Ackman and Crair, 2014) suggesting that early connectivity and function is substantially different from later development. In particular, early activity differs from the adult due to the presence of long periods of network silence and the presence of strong network oscillations (Luhmann et al., 2016), giving the early cortex the appearance of extreme synchronization. Acute slices display waves of synchronized activity during a limited developmental period (Ben-Ari et al., 1989; Moody and Bosma, 2005; Allène et al., 2008) that result from unique circuit configurations (Dupont et al., 2006; Bonifazi et al., 2009). *In vivo*, early cortical activity is characterized by bursts of rapid oscillations that synchronize multi-unit firing (Yang et al., 2009; Brockmann et al., 2011; Minlebaev et al., 2011). Calcium imaging *in vivo* shows hypersynchrony in superficial layers relative to mature patterns (Golshani et al., 2009; Rochefort et al., 2009; Siegel et al., 2012).

While these previous studies give an impression of cortical hyper-synchrony during map formation, they lack cellular resolution, do not sample the cortical depth and/or lack temporal resolution to determine spike correlations within a cortical column. We therefore used multi-electrode array recordings combined with spike-sorting of units measured throughout the depth of individual cortical columns to measure columnar synchronization in the developing visual cortex, a region for which the primary drivers of developmental activity and their role in circuit formation are largely known (Huberman et al., 2008; Ackman and Crair, 2014). During initial circuit formation (before eye-opening), activity in visual cortex is driven by spontaneous waves of activity in the retina (Ackman et al., 2012; Siegel et al., 2012). These are amplified and shaped into oscillations by the unique properties of thalamic and cortical circuits (Weliky and Katz, 1999; Hanganu et al., 2006; Murata and Colonnese, 2016). These early network properties are replaced by the mature cortical circuit dynamics when true vision develops around eye-opening (Rochefort et al., 2011; Colonnese, 2014; Hoy and Niell, 2015; Smith et al., 2015). We, therefore, examined statistical properties of local network interaction during the first three post-natal weeks, when cortical activity patterns are changing most rapidly. We asked whether: (1) activity driven by retinal waves is indeed hyper-synchronous relative to post-eye opening, supporting a refinement model for cortical columnar connections; or whether (2) this activity is similar to (or even less synchronous than) post-eye opening. The latter would support a constructionist model, suggesting that early spontaneous activity does not consist of synchronous bursts, but rather windows of local activation allowing adult-like network dynamics to drive the formation of neural assemblies.

## Materials and Methods

### Animal Care

Animal care and procedures were in accordance with *The Guide for the Care and Use of Laboratory Animals* (NIH) and approved by the Institutional Animal Care and Use Committee at The George Washington University. Postnatal day (P)0 is the day of birth. C57BL/6 were obtained from Hilltop Lab Animals (Scottsdale, PA, USA) as timed pregnant females, and kept in a designated, temperature and humidity-controlled room on 12/12 light/dark cycle.

### *In Vivo* Electrophysiology

Recording techniques have been previously reported (Shen and Colonnese, 2016), and are reproduced here for clarity. Carprofen (20 mg/kg) saline was injected 1 h prior to surgery to reduce pain and inflammation. Surgical anesthesia was induced with 3% isoflurane vaporized in 100% O_2_, verified by tail-pinch, then reduced to 1.5%–3% as needed by monitoring breathing rate. A vented warming table (36°C, VetEquip, Livermore, CA, USA) provided thermoreplacement. For attachment of the head-fixation apparatus, the scalp was excised to expose the skull, neck muscles were detached from the occipital bone, and the membranes were removed from the surface of the skull. Topical analgesic was applied to the incision of animals older than P8 (2.5% lidocaine/prilocaine mix, Hi-Tech Pharmacy Co., Inc., Amityville, NY, USA). Application to younger animals was lethal and discontinued. The head-fixation apparatus was attached to the skull with grip cement (Dentsply, Milford, DE, USA) over Vetbond^TM^ tissue adhesive (3M). Fixation bar consisted of a custom manufactured rectangular aluminum plate with a central hole for access to the skull. After placement, the animal was maintained with 0.5%–1% isoflurane until the dental cement cured, after which point it was allowed to recover from anesthesia on the warmed table.

For recording, animals were head-fixed and body movements were restricted by placement in a padded tube. Body temperature was monitored via thermometer placed under the abdomen and maintained between 33C and 36C via thermocouple heating pad (FHC, Bowdoin, ME, USA). Body motion was monitored with a piezoelectric device placed below the restraint tube. For electrode access, a craniotomy was performed thinning the skull if necessary and resecting small bone flaps, to produce a small opening (~150–300 μm diameter). Primary visual cortex was targeted by regression of adult brain lambda-bregma distances: 1.5–2.5 mm lateral and 0.0–0.5 mm rostral to lambda. All recordings were made using a single shank, 32 channel array arranged in two parallel lines of contacts (A1x32-Poly2-5mm-50s-177, NeuroNexus Technologies, Ann Arbor, MI, USA). The electrode penetrated the brain orthogonally to the surface and advanced to a depth of 750–1000 μm using a micro-manipulator (Narishige, Japan) until the top channels touched the dura. Isoflurane was withdrawn and the animal was allowed to acclimate inside the setup for at least 80 min prior to recording. Following 20 min of visual stimulation the spontaneous recording reported here lasted 30 min. All recording was performed in the dark (<0.01 Lumens). In animals older than P8, recording localization in monocular V1 was confirmed by the presence of a contralateral visual response to whole-field light flash that had the earliest response in layer 4. Ipsilateral visual local field potential (LFP) responses less than 10% of the contralateral response were also required. All animals were sacrificed by anesthetic overdose followed by decapitation. Brains were immersion-fixed in 4% paraformaldehyde for confirmation of electrode location.

### Data Acquisition and Analysis

Data was acquired at 32 kHz using the Digital Lynx SX acquisition system and Cheetah version 5.6.0 (Neuralynx, Inc., Bozeman, MT, USA). Signals were band-passed 0.1 Hz 9 kHz and referenced to a sub-cortical contact in at the bottom of the array. Analysis utilized custom MATLAB (MathWorks, Natick, MA, USA) routines and the open-source *Klusta*suite for spike isolation, clustering and manual curation (Rossant et al., 2016). A spike isolation strong threshold of 6 SD and weak threshold of 3 SD were needed to minimize low amplitude unclusterable spikes. Initial clustering was evaluated for merging or splitting of clusters based on visual waveform analysis and similarity. After eliminating clusters composed of noise, clusters with no modulation of autocorrelation near 0 ms and/or clear superposition of two or more distinct waveforms that could not be separated were marked as multi-unit clusters. The remaining, potentially single-unit, clusters were evaluated for inclusion as good single-units if the interspike interval (ISI) refractory violations (<2 ms) accounted for less than 1% of spikes. All clusters were assigned a primary contact localization by determining the minima of the mean waveform. Spike-time was assigned by rounding the peak time to the nearest ms. The layer identity of each channel was made relative to L4 which was identified in an age-specific manner: After the emergence of visual responses on P8, L4 was identified as the channel with the shortest latency 300–500 μm below the surface; for P4–P7, which lack visual response, L4 was identified from spontaneous spindle-bursts as the lowest channel with visible rapid oscillations in the LFP (Colonnese and Khazipov, 2010). For thalamic recordings, only clusters originating from contacts with multi-unit visual responses to 100 ms light flashes presented to the contralateral eye were used. Otherwise, procedures were identical to the visual cortex.

Inactive periods (down-states) were identified by the method of Renart et al. (2010). A total multi-unit activity (tMUA) raster was created by summing spike occurrences of all multi and single-unit clusters. To identify down-states, the tMUA signal was smoothed (Gaussian kernel SD 50 ms); periods where this convolved signal was less than 10% of peak OR where the ISI was greater than 50 ms were marked as down-states. “Active” periods of duration less than 50 ms were reclassified as down-states.

For the calculation of “Event size” (Figure 3), only Active periods were considered, and the number of single-units with at least one spike in sliding windows of 20 or 100 ms was determined. Only windows with more than one spike were considered. Probability for events of a given size was calculated by dividing the number of windows with events of a given size, by the total number of windows. To calculate event size probability in animals with more than 12 good single units, event size was determined for a random selection of 12 units. This process was repeated 20 times, and the mean probability of these repetitions was used for that animal. To calculate the change in probability, spike-times were jittered within active periods by a random number drawn from the uniform distribution between −1000 ms and 1000 ms and the probabilities recalculated. Binary firing vectors (Figure 4) were calculated using the shared Matlab function as described (Okun et al., 2012). For calculation of the Raster-Marginal, spikes were swapped between good neurons as well as MUA but only for spikes in the same layer group (L2–4 or L5–6). Calculation of spike-rate correlation (Figure 5) followed the method of Renart et al. (2010). Each single-unit raster was convolved by a normalized kernel that was the sum of a rapid time-window (J, 20 ms SD Gaussian) and a negative longer window (T, 4xJ). This approximates the effect of jittering the spikes over the same window to remove distorting effects of slow co-modulations in spike-rate. For comparison with truly random firing with locally appropriate spike-rates, jittered spike-trains (±1 s within active periods) were calculated, spike-rate comodulation calculated, and this process was repeated 100 times to generate mean and 95% confidence interval. Population coupling (Figure 6) was computed by the method of Okun et al. (2015). A summed multi-unit raster was created from the MUA and single-units for L2–4 and L5–6 separately, convolved with a Gaussian kernel (10 ms SD) which was used for the spike-triggered average for each single-unit in the corresponding layer group. A normalized spike-triggered rate was made after calculating the Raster Marginal as described above, the peak amplitude of which is used to normalize each animal’s population coupling.

**Figure 1:**
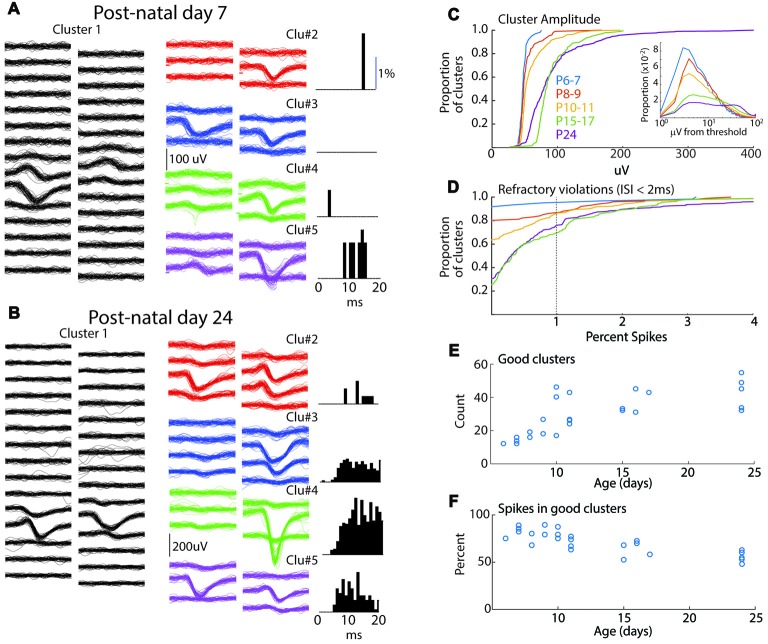
Spike-clustering in neonatal mice. **(A)** Five representative clusters from the same 7 day old animal (P7). Each trace is 1 ms, scale bar (100 μV) applies to all traces. On left (Cluster 1) show 50 traces from 30 channels in the poly-2 array (50 μm separation). Six surrounding channels are shown for four additional representative clusters in the middle, and the associated inter-spike interval (ISI) histogram (as measure of refractory period) for the same cluster is displayed at right. Low spike rates mean that ISIs can be used to eliminate obvious multi-unit clusters but are less reliable as positive evidence that clusters are single-unit. **(B)** Representative clusters for P24 animal. **(C)** Cumulative distribution of peak amplitude for all clusters (good and bad) in each age group used in the study. Insert shows spike amplitude distribution (all spikes including those not included in good clusters) as function of threshold for than recording. All ages show clustering near threshold, though a greater proportion of young animal spikes are <5 μV from threshold. **(D)** Cumulative distribution of the percent of refractory violations (ISI <2 ms) for all clusters. Note fewer violations in young animals. **(E)** Number of “good” clusters isolated for each animal by age. **(F)** Percentage of total recorded spikes that were placed in good clusters.

**Figure 2:**
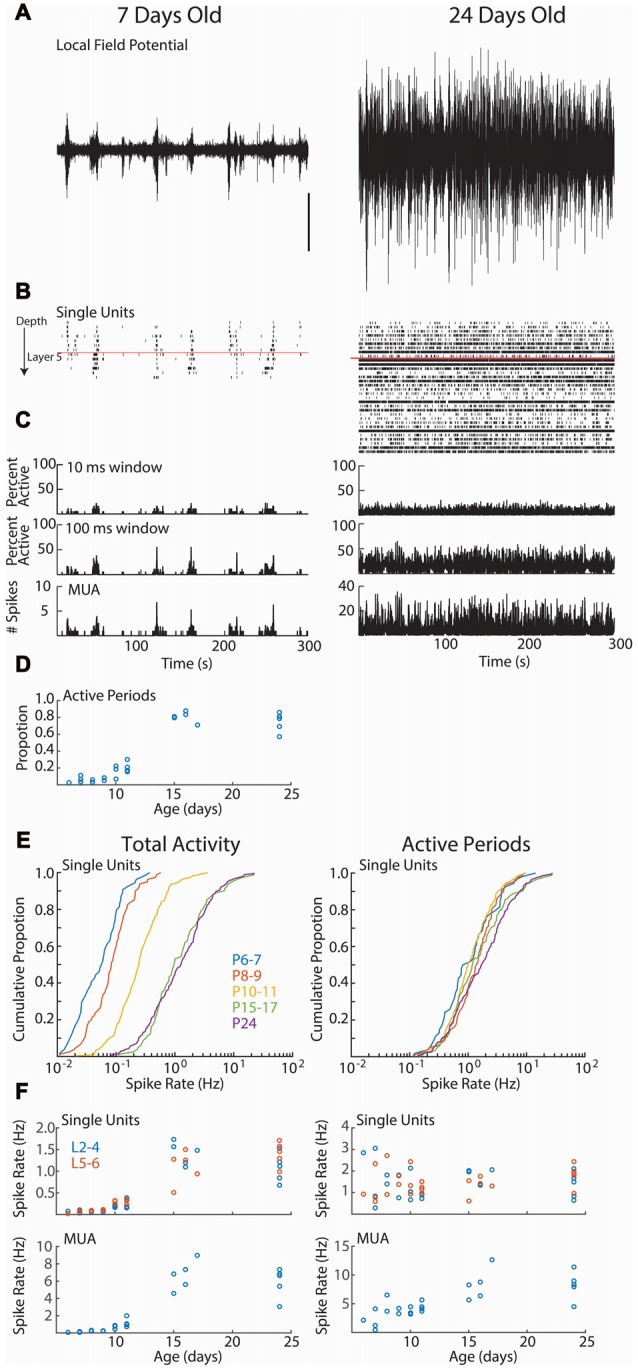
Cortical activity in neonatal rats is dominated by down-states. **(A–C)** Representative spontaneous activity (300 s) for a P7 and P24 rat. **(A)** Local-field potential from layer 2/3. Scale bar is 500 μV, and is for both traces. **(B)** Raster plot of all good single-units arranged by depth. Red line shows top border of layer 5. **(C)** Spike histograms showing the percentage of single-units active in a 10 ms (top) or 100 ms (middle) window. At bottom, the multi-unit activity (including spikes not sorted into good single-units) in 1 ms bins is displayed. **(D)** Proportion of recording occupied by active periods (see “Materials and Methods” Section) for each animal, by age (analysis of variance (ANOVA) for effect of age (groups P6–7, 8–9, 10–11, 15–17, 24), *df* = 4, *F* = 94.99, *p* = 10^−12^). **(E)** Cumulative distribution histogram of spike-rate of all single-units in each age group. Total activity is shown on the left, firing rate during active periods (down-states removed) is on right. Developmental changes in single-unit firing rate are due to increased prevalence of down-states at these ages. **(F)** Firing rates for single-units (above) and total multi-unit activity (tMUA) by age. Total activity (left) effect of age (L2–4 *F* = 53.85, *p* = 10^−10^; L5–6 *F* = 39.72, *p* = 10^−9^); MUA effect of age (*F* = 39.15, *p* = 10^−9^). Active periods only (right; L2–4 *F* = 1.40 *p* = 0.29; L5–6 *F* = 2.39, *p* = 0.08); Active period only MUA (*F* = 14.36, *p* = 10^−5^).

**Figure 3:**
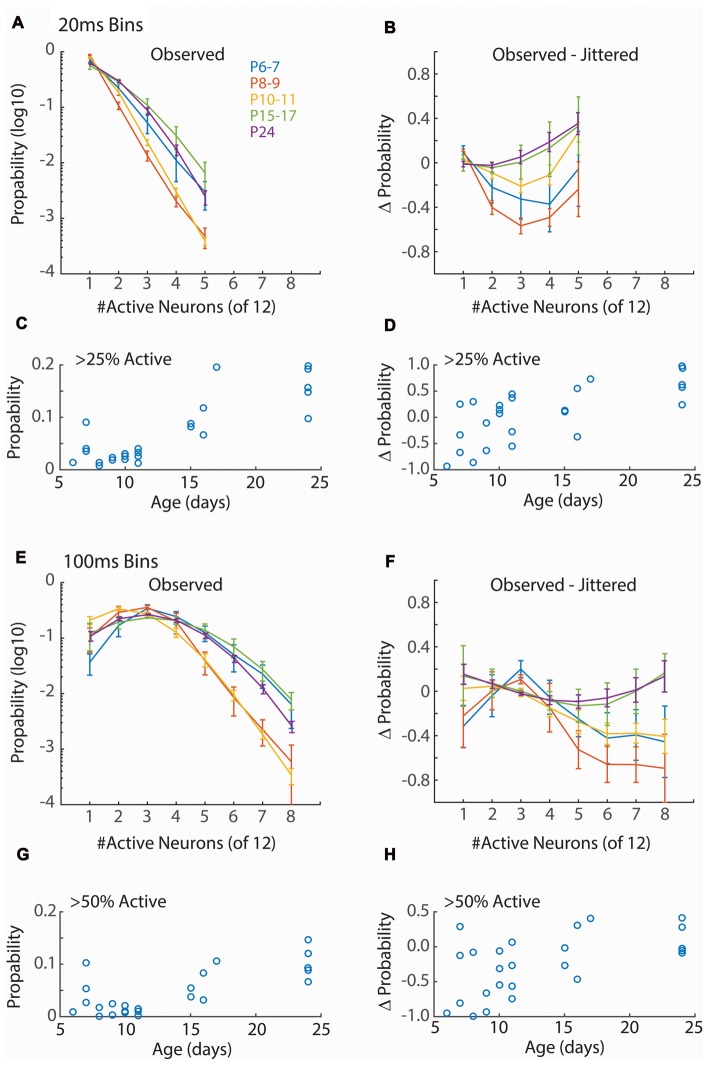
Synchronization of firing increases with age. **(A)** Distribution of neuronal event size for 20 ms windows. Points show mean and SEM of distributions for animals in each group. For each animal the probability of observing events of the indicated size in a 20 ms window from a random assignment of 12 neurons is shown. Only event sizes observed in all animals (<6 neurons) are analyzed. Only active periods are considered. ANOVA for effect of synchronization (*F* = 546.44, *p* = 10^−66^), Age group (*F* = 48.80, *p* = 10^−22^) and interaction (*F* = 8.00, *p* = 10^−12^). **(B)** Change in event size probability relative to jittered spike trains (Prob − Prob_jitt_)/(Prob + Prob_jitt_). Young animals (P6–11) have lower probabilities of synchronized events than expected by chance (ANOVA for synchronization *F* = 6.56, *p* = 0.0001; Age *F* = 10.79, *p* = 10^−7^; interaction *F* = 1.34, *p* = 0.19). **(C)** Proportion of 20 ms bins with more than 25% of single-units active for each animal by age. ANOVA for age group (*F* = 16.81, *p* = 10^−6^). **(D)** Change in probability (vs. jittered) for >25% synchronization by age (*F* = 2.18, *p* = 0.11). **(E)** As **(A)** but 100 ms window. Only event sizes observed in all animals (<9 neurons) are analyzed. Synchronization at P8–9 and P10–11, but not P6–7, is lower than juvenile ages. ANOVA for effect of synchronization (*F* = 152.67, *p* = 10^−66^), Age (*F* = 30.96, *p* = 10^−19^) and interaction (*F* = 8.51, *p* = 10^−19^). **(F)** As **(B)** but 100 ms window (Synchronization *F* = 4.84, *p* = 0.0001; Age *F* = 9.84, *p* = 10^−7^; interaction *F* = 1.59, *p* = 0.041). **(G)** As **(C)** but probability of events with >= 50% synchronization (*F* = 7.11, *p* = 0.001) of units are shown. **(H)** As for **(D)** but events >= 50% synchronization (*F* = 3.33, *p* = 0.03).

**Figure 4:**
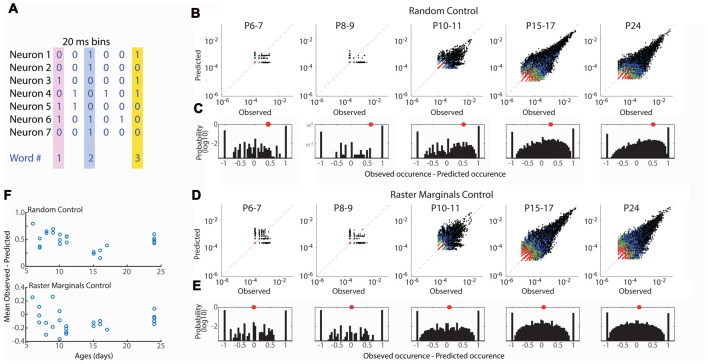
Developmental conservation of neuronal assembly properties. **(A)** Measurement of neuronal assemblies as unique “words” consisting of binary firing vectors for 20 ms windows. Only assemblies of 3+ units are considered. **(B)** Probabilities of observed word occurrence vs. occurrence predicted by random jittering of spikes. Graphs show all words in each age group. Black dots are 3 neuron words, blue 4, green 5, red >= 6. **(C)** Probability distribution for observed—predicted occurrence of all words in each age group. One means that word occurred only in observed data; −1 means it occurred only in jittered data. Distribution is right shifted at all ages, indicating greater occurrence of words than expected by chance throughout development. Red dot shows mean of distribution. **(D)** As **(B)** but predicted probability is calculated by Raster Marginal method (Okun et al., 2012) which controls for changes in network properties controlling the timing of spikes. **(E)** As **(C)** but for Raster Marginals control. **(F)** Mean difference of word distributions from random (top) or raster marginal (bottom) model for each animal by age. ANOVA for effect of age group (Random *F* = 8.35, *p* = 0.0004; Marginal *F* = 1.32, *p* = 0.297).

**Figure 5:**
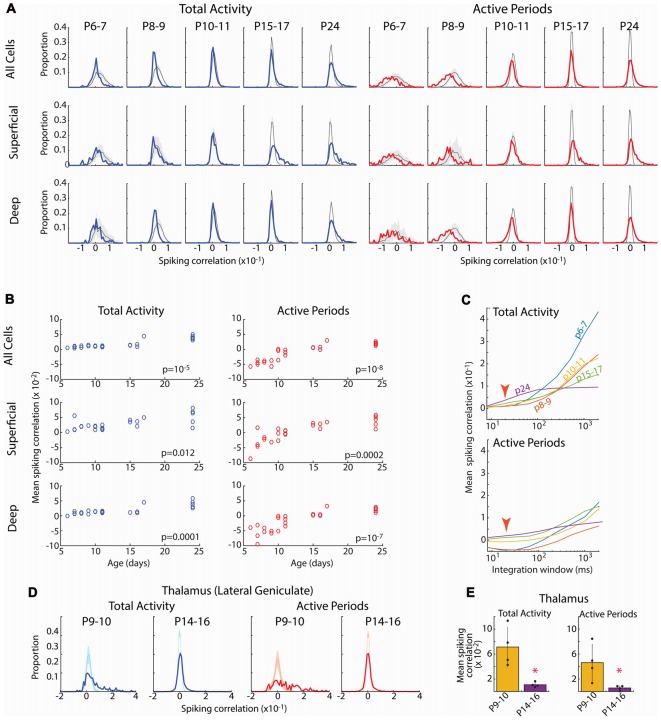
Developmental increase in pair-wise spiking correlations. **(A)** Distribution of pair-wise firing rate correlations for all neuron pairs in each age group. Blue line shows correlations for total activity including down-states; red line shows correlations when analysis is limited to active periods. Shading shows 95% confidence interval of jittered correlations. Top row shows all pairs, middle row shows superficial vs. superficial neurons only (L2–4), bottom row shows deep vs. deep neurons only (L5–6). Total activity shows more units with positive correlations than expected by chance after P15, but fewer correlated units P6–9. When inactive periods are removed, distributions shift left particularly at P6–7 and P8–9. **(B)** Mean correlations for each animal by age for the Total (left) and limited to Active periods (right) for each of the neuron populations. Listed *p*-values are for ANOVA effect of age group. **(C)** Effect of integration window (SD of Gaussian filter) on spiking correlations. Red arrow shows integration window used in **(A)** (20 ms). Developmental increase in mean correlations for total activity reverses at large integration windows suggesting that early activity is co-modulated by slow oscillations (likely retinal waves) but within such activations, it is poorly correlated. **(D)** Distribution of pair-wise firing rate correlations for neurons in lateral geniculate nucleus (visual thalamus) for select ages. During the period of retinal waves, thalamic neurons are positively correlated for both all activity and when analysis is limited to active periods. **(E)** Mean and standard deviation of mean correlation for each pup (*n* = 4). *p = 0.02 by rank sum test.

**Figure 6:**
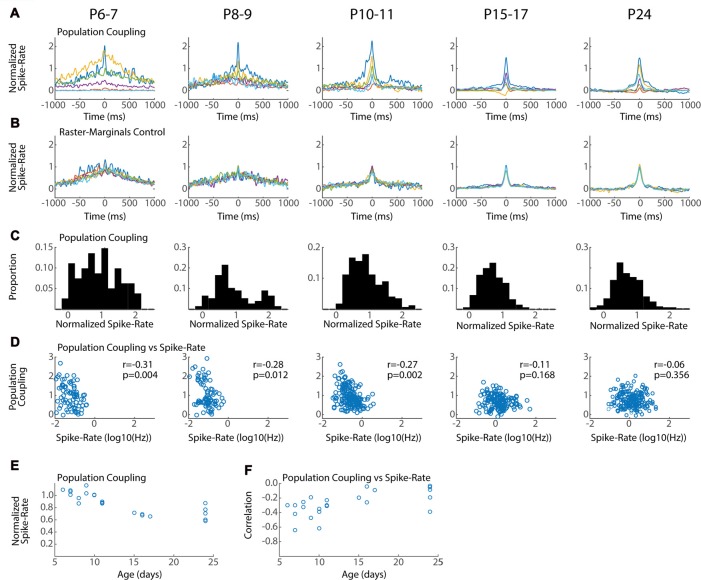
Conservation of local network integration during development. Population coupling (normalized spike-triggered multi-unit firing rate) is correlated with local connectivity in adults (Okun et al., 2015). **(A)** Representative population couplings for five neurons from a single animal in each age group. Each animal has neurons with diverse coupling, though the temporal specificity of the coupling increases with age. **(B)** Coupling for the same neurons after randomization (Raster-Marginals). **(C)** Distribution of population coupling for all single-units in the age group. **(D)** Spike-rate plotted against population coupling for all neurons in each age group. Spike-rate changes between ages do not strongly affect results, though the presence of very low spike rate appears required for high population coupling. Correlation (r) between spike-rate and population coupling, and the *p*-value of this correlation (p) are shown for each age group. **(E)** Mean population coupling for each animal by age. ANOVA for effect of age group (*F* = 11.24, *p* = 0.00008). **(F)** Mean correlation of coupling vs. spike-rate for each animal by age. ANOVA for effect of age group (*F* = 3.99, *p* = 0.016).

### Statistics

All statistical tests are described in the results along with *p*-values. *P*-values below 0.001 are rounded to the nearest power of 10.

## Results

We examined spontaneous activity in monocular visual cortex of unanesthetized, head-fixed mice. Multi-unit and LFP analysis of this same data-set has been previously reported (Shen and Colonnese, 2016). Recordings targeted five key developmental age groups: P6–7 and P8–9 during the period of cholinergic retinal waves, when topography and eye specificity is established; P10–11 during the period of glutamatergic retinal waves; P15–17 after eye-opening, during the pre-critical period (Smith and Trachtenberg, 2007) when cortical state modulation of spontaneous activity has emerged; and P24 during the critical period for ocular dominance plasticity when mature cortical dynamics are largely in place (Hoy and Niell, 2015). Recordings from animals at P4 and 5 yielded fewer than 10 clusters per animal (mean 7 ± 3 SD), which was considered insufficient for network analysis.

Network analysis was made by isolation of presumptive single-units from a single shank, dual column multi-electrode array placed perpendicular to the cortical layers, allowing for simultaneous recording from layer one though the top of layer six. Sorting using the masked EM algorithm (Rossant et al., 2016) was of similar quality between age groups (Figure 1). Spike amplitudes in the youngest age group were lower and clustered near threshold, but overall the similarity of spike waveforms placed in the same cluster was comparable between age groups (Figures 1A,B). Refractory violations (ISIs < 2 ms) were rarer in young animals, likely as a result of lower spike-rates (Figure 2), making this a less reliable index of cluster quality in neonates. The number of well isolated (“good”) clusters extracted from each animal increased rapidly with age (Figure 1E). This was not a result of more clusters or spikes rejected in young animals. In fact, the proportion of total recorded spikes that were placed in good clusters was highest during the first 2 weeks (Figure 1F). In general, while the lower size and reduced ability to use refractory violations to separate clusters increases the chances of multi-unit clusters, the reduced spike detection counteracts this effect. We are confident that clustering effectively enriches for single neurons in all age groups. Because analysis of fewer neurons could bias results between age groups, when appropriate, analyses were performed on a random selection of 12 units (the minimum number of good clusters isolated), calculating the relevant metrics, and repeating this process to generate a mean for the animal. Consistent with MUA (Shen and Colonnese, 2016) fewer deep layer single-units were isolated in P6–7 animals (ratio 1.5 superficial/deep) than in older animals (P8–9 0.40; P10–11 0.48; P15–17 0.46; P24 0.56).

We first asked whether age is associated with differences in the firing rates of individual neurons (Figure 2). As previously shown for unanesthetized mice (Adelsberger et al., 2005; Ackman et al., 2012; Shen and Colonnese, 2016) and rats (Hanganu et al., 2006; Colonnese, 2014), activity during the first 2 weeks post-natal is dominated by lengthy periods of network silence (“down-states”). The down-states are interrupted by periods of activation driven by spontaneous retinal waves (P2–9) and then by retinal waves plus activity generated spontaneously within the cortex (Colonnese and Khazipov, 2010). Down-states lasting more than 200 ms disappear around eye-opening in rats (Colonnese, 2014) and mice (Shen and Colonnese, 2016). Single unit activity showed a similar pattern, being almost completely restricted to 2–10 s active periods occurring every 30–60 s at P6–11. Extended periods of network silence were rare in the P15–17 or P24 groups (Figure 2D).

Total single-unit spike-rates at P6–7 and P8–9 were at least an order of magnitude lower than juvenile rates (Figures 2E,F, left), with P10–11 rates intermediate. However, if the down-states are removed and only periods of activation considered, then single-unit spike-rates were not significantly different between ages (Figures 2E,F, right), suggesting that active periods for spontaneous activity in young and old mice are similar, and that age differences in firing result from the prevalence of down-states during the first two post-natal weeks. For comparison with previous non-clustered recordings (Colonnese et al., 2010; Shen and Colonnese, 2016), we present tMUA spike rates (which additionally includes spikes not placed in good clusters). MUA spike rates show significant increases both for total activity and during active periods, particularly between P11 and P14 (Figure 2F, bottom).

### Development of Network Synchronization and Composition

Functional hyper-connectivity of inputs and/or local connections due to either increased connectivity or lack of desynchronizing inhibition is expected to result in activity events with high participation rates, as has been observed by calcium imaging of layer 2/3 *in vivo* (Rochefort et al., 2009; Siegel et al., 2012). Because firing during early ages is largely restricted to the troughs of spindle-burst oscillations we first measured event participation in 20 ms windows, approximately the window of firing during these early oscillations (Hanganu et al., 2006; Colonnese and Khazipov, 2010). To measure columnar event participation, we calculated the percentage of single-units active (at least 1 spike) in sliding 20 ms windows. Event participation rates show clearly that early activity is not hyper-synchronous (Figure 3A). In fact, participation rates in the youngest group (P6–7) is similar to juvenile (P15–17 and P24), and activity is less synchronous at P8–9 and P10–11 before achieving stable values by P15 (Figure 3C). Event participation is affected by spike-rate as well as spike timing. To control for the former, we calculated the change in event probability after jittering spike times by a random amount between ± 1 s within active periods and calculating a deviation index (Prob − Prob_jitt_)/(Prob + Prob_jitt_). This analysis showed that event participation during the periods of retinal waves (P6–11) were actually lower than expected from random firing, while the juvenile synchrony is slightly higher than expected (Figures 3B,D).

Currents in young neurons have longer decay times, potentially increasing the integration time and tolerance for synchrony. We therefore examined a longer time window for neural events (100 ms) which encompasses a complete cycle of the early oscillations. Event size was larger for 100 ms windows, as expected, but the relative synchronization of firing between ages was similar to 20 ms windows (Figures 3E–H). Thus, regardless of window size, event participation rates of neonates are lower than those of juveniles and lower even than expected by chance given neuronal firing rates. This suggests that activity in the neonatal cortex is actively decoupled.

Our event participation data reject the hypothesis that mature neuronal ensembles are formed by fractioning larger ensembles but obscure the specifics of which neurons fire together. To understand the development of neural ensembles we used an analysis of the occurrence of unique binary spike vectors, or “neural words” (Fiser et al., 2004; Figure 4A). The occurrence of specific words is tested against the distribution expected by random firing, modeled here by random jittering the occurrence of spikes ± 1 s. We examined word distributions for words three neurons or longer in 20 ms windows. As expected given the larger number of good clusters in older animals, the total number of observed words increased with development, however word occurrence was greater than expected for random activity at all ages (Figures 4B,C). The mean of the observed probability-predicted probability (by chance) was larger in in P6–11 animals than P15–17 and P24 (Figure 4F). This relationship could indicate that connectivity in the young network is more strongly non-random than juvenile, or that the network property which synchronizes spike-timing regardless of ensemble participation is stronger in young animals (Okun et al., 2012), consistent with the presence of spindle-burst oscillations during this time period. To distinguish between these possibilities we predicted word occurrence distributions using the Raster Marginal method (Okun et al., 2012), which swaps spikes between clusters, thereby keeping intact temporal restraints on spike-timing but randomizing occurrences within clusters. Mean observed-predicted occurrence using the marginal control was near or below zero for all ages, and not different between age groups (Figures 4D–F), suggesting that at all ages there exist fewer functional interconnections between neurons than would be expected by chance.

### Neural Firing Rate Correlations Increase During Development

Spike-rate correlations reflect neural connectivity filtered through cellular, synaptic and circuit properties influencing synchronization, particularly inhibition (Renart et al., 2010; Helias et al., 2014). Ineffective inhibition, neuronal hyperexcitability and hyperconnectivity would be expected to increase spike-rate correlations in infant cortex, as has been observed for calcium signal in somatosensory cortex (Golshani et al., 2009). To test this we calculated the distributions of pair-wise spike-rate correlations for all good units in an animal. Spike-rate correlations were evaluated by quantifying spike-rate comodulation within a rapid time window (*T* = 20 ms full width at half amplitude Gaussian) corrected for slow modulation of firing rates (*J* = 80 ms; Renart et al., 2010). Pairs were also subdivided into superficial (L2–4) and deep (L5–6) to assay the development of local vs. total synchronization. Because the presence of down-states, which increase correlations by enforcing silence in almost all neurons, changes dramatically during development, we examined correlation distributions for total activity as well as when restricted to active periods.

The spike correlation distributions for total activity were grossly similar at all ages (Figure 5). The large majority of pairs had correlations near zero, but the distribution evidenced a right-ward shift toward a larger proportion of correlated neuron pairs than expected by chance (jittered spikes) that grew stronger with age. As a result, the mean spike correlation of each animal increased when measured between all neurons, as well when restricted to deep or superficial neurons (Figure 5B). Limiting the analysis to active periods caused a leftward shift in the correlation distribution at all ages. This shift was much larger for neonatal animals, even resulting in mean correlations below zero for the P6–7 and P8–9 groups. Thus at these youngest ages, firing rates are less correlated on a short time-scale (20 ms) than expected by chance, suggesting inhibitory or other another desynchronizing element is powerful even during early development. As a result of the negative mean correlations in neonates, developmental increases in mean correlation were significant for total, superficial and deep neurons (Figure 5B).

Calcium imaging studies have described developmental desynchronization of layer 2/3 activity (Golshani et al., 2009; Rochefort et al., 2009), opposite from the increasing synchronization we observed here. While multiple factors could contribute to this difference, the effective integration window for firing is a prominent difference between our spike-rate correlations and calcium imaging. To examine the role of integration window on mean spike-rate correlations, we systematically varied *T* (with *J* also increasing at 4*T). This showed a strong, age dependence of correlation on integration window (Figure 5C). For total activity, as integration windows approach 400 ms the developmental relationships between neonatal and juvenile animals reverse, with activity at P6–11 becoming more synchronous than P15–17 and P24. Thus during the period of retinal waves, neurons show co-modulation of firing rates on the time scale of these waves, but synchronization within waves is lower than or similar to juvenile levels. When correlations were limited to active periods, integration window does not have as dramatic an effect. However, negative mean correlations present in neonates were only present when *T* was less than 300 ms. To determine if this dependence of integration window was the result of changing *T* or *J*, we varied *J* while keeping *T* constant. Correlations were largely unchanged out to a *J* of 3 s (data not shown), beyond the duration of a single retinal wave (Blankenship and Feller, 2010; Ackman et al., 2012). Finally, because maximal negative mean correlation is inversely proportional to the number of neurons, we recalculated pair-wise correlations for *N* = 12 neurons in all groups (data not shown). Mean correlations were not significantly different in this *N* limited case, showing that the growth of correlation with age is a true effect of development, not of the number of neurons isolated by spike-sorting.

Such low correlation between cortical neurons is surprising given that the contacts sit within a single topographic locale and neuron firing in all layers is temporally limited to the troughs of spindle-burst oscillations (Colonnese and Khazipov, 2010). To determine the effect of retinal waves and synaptic refinement on correlation in a structure with known refinement of input connectivity (Chen and Regehr, 2000; Ziburkus and Guido, 2006), we analyzed spike-rate correlation in the region of the lateral geniculate nucleus of the thalamus responsive to the contralateral eye. In contrast to the cortex, LGN neurons at P9–10 when poly-innervation by retinal ganglion cells is high, were largely positively correlated both with total activity as well as when analysis was limited to active periods only (Figures 5D,E). By P14–16, when poly-innervation is reduced, correlations become centered around zero, similar to cortex at the same age. Thus in a structure with demonstrated refinement of connectivity, pair-wise spike correlations show heightened synchronization.

In total, while the pair-wise firing rate correlations in the cortex are sensitive to integration window, within physiologically relevant time intervals for spike integration (10–300 ms), they are robust and consistent with increasing connectivity driving synchronization of the neural activity during cortical development. Combined with event participation, firing rate correlations suggest that early networks contain decorrelating influences that keep synchronization below that expected by chance given firing rates and patterns.

### Characteristics of Local Network Integration Are Established Early in Development

Neurons vary in their local vs. distal connectivity, a feature that is correlated with the degree to which their firing is coupled to mean firing rates in the local network, called “population coupling” (Okun et al., 2015). We hypothesized that hyper-connectivity during early development would result in increased population coupling and fewer neurons with activity that is independent of local firing (so called “soloists”). To test this we calculated the spike-triggered multi-unit activity for each good unit. Triggered spike rates are converted to standardized “population coupling” by normalization to the same measure for spikes shifted using the Raster Marginal method, thereby constructing a mean coupling for the animal that can be used to normalize between groups. The temporal characteristics and signal-to-noise of population coupling changed dramatically between P6–7 and P15–17, but all ages showed dramatic variance in the absolute degree of coupling. Every animal had neurons with strong coupling as well as neurons with little or even negative coupling (Figure 6A). The total distribution of normalized spike-rates showed a similar pattern of diverse coupling in each age group, with a peak between 0.5 and 1 (Figure 6C). The three youngest age groups (P6–11) had an additional population of highly coupled neurons that was not apparent in the older ages. As a result at these ages population coupling was negatively correlated with spike rate (Figure 6D) and mean coupling was reduced with age (Figure 6E). By P15, population coupling is not correlated with spike-rates similar to adults (Okun et al., 2015).

In total our results show that while population coupling is elevated for a sub-population of low-firing neurons during early development, neurons with weak and strong population coupling exist even in the youngest networks. These results suggest that a neuron’s relative local integration is established early and maintained throughout development.

## Discussion

Here we used multi-electrode array based spike-sorting in very young, head-fixed mice to quantify features of local network interaction within a visual cortical column during the period of spontaneous retinal waves and compared these to spontaneous activity in the week after eye-opening, when many aspects of ongoing cortical activity emerge (Rochefort et al., 2009, 2011; Hoy and Niell, 2015). Our primary finding is that—despite the macro-patterning of activity present in cortex during the period of retinal waves, in which long silent periods are interrupted by large oscillations that control firing times (Hanganu et al., 2006; Colonnese and Khazipov, 2010), suggesting hypersynchrony of the developing network—the firing of cortical neurons is remarkably uncorrelated and adult-like patterns of network interaction are achieved remarkably early, either during the period of glutamatergic retinal waves or immediately after eye-opening. Our results support a constructionist model of vertical circuit formation in cortex, rather than one of exuberant connectivity followed by refinement, and suggest that retinal waves provide activity remarkably more similar to adult activation than might be expected.

### Calcium vs. Action Potentials in Network Analysis and Function

Our results differ from *in vivo* calcium imaging studies, which indicate a decrease in correlation between layer 2/3 neurons (Golshani et al., 2009; Rochefort et al., 2009; Siegel et al., 2012), likely because of the time course over which synchronization is measured. Integrating spike-rates on the timescale of calcium indicators selectively increased the pair-wise correlations of young neurons (Figure 5C). Current-clamp recordings suggest that early hyper-synchronization observed in imaging is due to increased firing probability during the “up”-states of the slow-wave (Golshani et al., 2009; Colonnese, 2014), and not co-participation of neurons in local ensembles, which appears to grow (Berkes et al., 2011) or remain similar (Figure 3) during development.

One important caveat to the current results is that the increased temporal and single-spike resolution of electrophysiology comes with the inherent ambiguity of spike-sorting. The smaller transmembrane currents of young neurons make action-potentials less likely to be detected on multiple electrodes and reduce waveform variability, potentially compromising sorting. Current spike-sorting quality metrics emphasize separation and reduction of over-splitting (Hill et al., 2011) and the application of these metrics to the high-dimensional space of large arrays is not standard (Harris et al., 2016; Rossant et al., 2016). Confirmation that the spikes in a single cluster originate from a single neuron relies on visual confirmation of waveform and elimination of clusters with high rates of refractory period violation. The low spike-rate of young animals should cause an increase in false negatives (failure to reject poly-neuronal clusters). Thus while spike waveform consistency was similar between ages (Figure 1), it remains possible that more “good” clusters in young animals contain multiple neurons, which may explain some of the increase in number of neurons isolated with age. The developmental growth in correlations is unlikely to result solely from poor sorting, however, as it is also evident in multi-unit activity measured at similar ages (Berzhanskaya et al., 2017).

Another potential issue is the necessary sparse sampling of neurons by our arrays and the under-sampling of neurons in young tissue as a result of their smaller action potentials. This under-sampling appears to result in our detection of fewer neurons in P6–11 animals. Imaging studies indicate that close to all L2–3 neurons make calcium events at these ages (Siegel et al., 2012), so it is unlikely that there is large population of silent neurons that comes “online” only after eye-opening. Current-clamp recordings find action potentials in a large majority of neurons recorded (Colonnese, 2014), supporting the possibility that the low number of neurons observed is simple failure to pick up small action potentials from distant neurons. We have tried to correct as best as possible for the computational biases of this under-sampling by limiting the network analyses in older animals to subgroups of the same number of neurons as in young. Using the same methods in thalamus, we were able to detect clear reductions in synchronization, suggesting that similar changes would have been apparent in cortex if they were present.

Finally, it is not clear whether action potentials or calcium fluxes are the relevant components for plasticity. Retinal waves induce plateau potentials in cortical neurons (Colonnese, 2014) which both suppress action potentials and may cause neurotransmitter release. Thus the poor synchronization of action potentials may be compensated by synchrony of calcium transients and sub-threshold depolarization.

### Constructionism vs. Refinement

Our results support a “constructivist” model of intra-columnar cortical development in which correct connections, informed by guidance molecules and confirmed by activity, are largely made early, without large-scale elimination of incorrect connections (Quartz and Sejnowski, 1997; Katz and Crowley, 2002). We observed no reduction in event participation, word distributions, or pair-wise correlations, which would be expected if the network experienced a period of widespread functional hyper-connectivity followed by synapse elimination. This does not mean that inappropriate synapses are not formed and eliminated within cortical columns. Rather, it means inappropriate synapses are a minority of total synapses, and play little functional role. Map formation in the cortex may be different from sub-cortical regions such as thalamus and superior colliculus that are the initial recipients of topographic connections. These regions do incur periods of exuberant synaptic connectivity (Chen and Regehr, 2000; Lu and Constantine-Paton, 2004; Ziburkus and Guido, 2006), and we actually observed a reduction in firing rate correlations in LGN while they were increasing in cortex. Our results are consistent with multiple findings that thalamocortical and cortico-cortical connectivity is refined very early (Chiu and Weliky, 2002; Katz and Crowley, 2002; Ko et al., 2013; Yang et al., 2013). Our results expand upon these by showing that within topographically aligned columns functional connectivity is sparse even during initial map formation. Early and consistent refinement may be specific to intra-columnar circuits, which reflect a single topographic location, while horizontal connectivity may be subject to different rules. Studies have observed both increases and decreases in horizontal synchronization during development (Callaway and Katz, 1990; Fiser et al., 2004; Minlebaev et al., 2011).

One of our more unexpected findings is that variance in population coupling is present from as early as intra-cortical synapses are present and can drive activity locally (Blue and Parnavelas, 1983; Valiullina et al., 2016). While we cannot prove that early “soloists”, neurons with low population coupling (Okun et al., 2015), are the same neurons that become adult soloists, our data suggest that this identity is set early, even before many of the long-range connections that will drive soloists are formed. The existence of a population of low-firing neurons with high-population coupling at P5–9 is at first glance contradictory to the pair-wise spike-rate correlations, which show lower local correlation at these ages. We suspect this early high-coupling, which occurs on a much slower time course than coupling in adults, exists because of the massive modulation of all activity in visual cortex by the slow retinal waves. This is reflected in the net positive jittered pair-wise correlations (Figure 5A gray lines). Thus, in total, our results are consistent with a model of early activity in which retinal waves increase firing rates globally, but the microstructure of correlations within this activation window is actually lower than for post-wave “spontaneous” activity. It is possible that the low-firing highly-coupled neurons are the youngest neurons which have not integrated into the local network, but still receive shared input from thalamus or widely distributed but weak local connectivity.

### Mechanisms of Activity-Dependent Development

While much is still poorly understood about the process leading to the formation of refined cortical ensembles, our data clearly indicate they do not emerge from larger, less refined functionally connected groups of neurons. In fact, in the youngest animals (P6–7 and P8–9) neuronal firing is actually less synchronous than would be expected by chance. This was true for both the participation rates as well as pair-wise correlations. Reduced spike-fidelity in young animals (Valeeva et al., 2010), combined with corticocortical connectivity, both electrical and chemical, that is very low (Yu et al., 2012), predict correlations near chance. However, the negative correlations require a desynchronizing element to decorrelate activity driven by the massively synchronous spindle-burst oscillations coming from thalamus (Helias et al., 2014). During this limited early period, thalamic axons synapse on inhibitory subplate neurons (Kanold and Luhmann, 2010) as well as somatostatin neurons in layer 5 (Marques-Smith et al., 2016; Tuncdemir et al., 2016) before shifting to their adult targets, providing one possible mechanism of inhibitory desynchronization. An implication of this anti-correlation is that the net effect of correlation based plasticity should be toward the elimination of new synapses, a phenotype observed in superior colliculus for the same ages (Colonnese and Constantine-Paton, 2006). In fact the only demonstrated synaptic plasticity *in vivo* during this time period is the elimination of poorly coordinated and ineffective synapses (Winnubst et al., 2015).

### Circuits, Synapses and Synchronization: The More they Change the More they Stay the Same

Developing cortical networks undergo remarkable changes in the amount and pattern of neural activity (Khazipov et al., 2013). In visual cortex, a large majority of the changes occur in rapid succession around eye-opening, though they are not strongly dependent on patterned vision. At this time immature spindle-burst synchronization of neural firing ends, cortical waking and sleep states emerge, thalamic amplification of retinal input is down-regulated, and the capacity of the circuit to follow relevant high frequencies emerges (Colonnese et al., 2010; Rochefort et al., 2011; Colonnese, 2014; Hoy and Niell, 2015; Shen and Colonnese, 2016). Somatosensory cortex makes a similar shift, though 4 days earlier, perhaps because whisking starts earlier than eye-opening (Colonnese et al., 2010; Minlebaev et al., 2011). Human infants undergo a similar shift 2–4 weeks before term (Tolonen et al., 2007; Colonnese et al., 2010; Fabrizi et al., 2011; Chipaux et al., 2013). The synaptic and network mechanisms of this shift are unknown, though they likely involve increased action potential threshold, development of ascending neuromodulators and functional integration of GABAergic interneuron subtypes, particularly those mediating fast-feedforward inhibition (Luhmann and Prince, 1991; Daw et al., 2007; Golshani et al., 2009; Colonnese, 2014). Inhibition is a controlling factor in many developmental transitions, particularly the onset of ocular dominance plasticity, leading to the suggestion they are “master” regulators of development, transforming activity in order to switch function (Le Magueresse and Monyer, 2013). We observed remarkable stability of the network properties between P10–11 and P15–16, ages between which feedforward inhibition develops in visual cortex (Colonnese, 2014). Thus, our results suggest an alternate framework, which is that inhibitory (among other) development occurs to maintain firing-rate and synchrony homeostasis in the face of increasing synaptic density and its inherent excitability (Hengen et al., 2013). By this model, interneuron integration occurs not as a developmental program to transform activity, but as a bulwark against increasing activity resulting from excitatory synaptogenesis. Changes in the pattern of neuronal oscillations occurring at the same time may, in fact, be side-effects of the circuit changes obscuring the deeper similarity between early and late ages.

One conclusion of this homeostasis theory of network synchronization is that retinal-wave activity (which dominates P6–11 firing) does not drive unique early ensembles of hypersynchronous firing in order to induce wiring of a single column, a conclusion also observed by L2/3 calcium imaging (Siegel et al., 2012), but rather to model adult cortical activity. It should be noted that the maintenance of correlational structure does not imply that individual neurons maintain connections across development. In fact, mature local ensembles form by rearranging specific connections while maintaining the same total connectivity after eye-opening (Ko et al., 2013). Our result shows that despite large-scale changes in the factors that regulate synchronization in adults, network properties in young networks are maintained so that the firing correlations caused by early connectivity can be read out and modified to drive circuit formation and “refinement”.

## Author Contributions

MTC designed the experiments, analysis and wrote the article. JS designed and performed analysis. YM performed experiments.

## Conflict of Interest Statement

The authors declare that the research was conducted in the absence of any commercial or financial relationships that could be construed as a potential conflict of interest.
